# MRI evaluation of tibial tunnel wall cortical bone formation after platelet-rich plasma applied during anterior cruciate ligament reconstruction

**DOI:** 10.2478/raon-2013-0009

**Published:** 2013-05-21

**Authors:** Mitja Rupreht, Matjaž Vogrin, Mohsen Hussein

**Affiliations:** 1 Radiology Department, University Medical Center Maribor, Medical Faculty, University of Maribor, Slovenia; 2 Orthopaedics Department, University Medical Center Maribor, Medical Faculty, University of Maribor, Slovenia; 3 Artros Center for Orthopaedic Surgery and Sports Medicine, Ljubljana, Slovenia

**Keywords:** anterior cruciate ligament graft, platelet-rich plasma, tibial tunnel, cortical bone, MRI

## Abstract

**Background:**

After anterior cruciate ligament (ACL) reconstruction, formation of cortical sclerotic bone encircling the femoral and tibial tunnel is a part of intratunnel graft healing. During the physiological cascades of soft tissue healing and bone growth, cellular and hormonal factors play an important role. The purpose of this study was to non-invasively but quantitatively assess the effect of intraoperatively applied platelet-rich plasma (PRP) on the formation of cortical bone encircling the tibial tunnel.

**Patients and methods:**

In fifty patients, standard arthroscopic ACL reconstructions were performed. The PRP group (n = 25) received a local application of PRP while the control group (n = 25) did not receive PRP. The proximal tibial tunnel was examined by MRI in the paraxial plane where the portion of the tibial tunnel wall circumference consisting of sclerotic cortical bone was assessed with testing occurring at one, two and a half and six months after surgery.

**Results:**

At one month after surgery, differences between the groups in the amount of cortical sclerotic bone encircling the tunnel were not significant (p = 0.928). At two and a half months, the sclerotic portion of the tunnel wall in the PRP group (36.2%) was significantly larger than in the control (22.5%) group (p = 0.004). At six months, the portion of sclerotic bone in the PRP group (67.1%) was also significantly larger than in the control (53.5%) group (p = 0.003).

**Conclusions:**

Enhanced cortical bone formation encircling the tibial tunnel at 2.5 and 6 months after ACL graft reconstruction results from locally applied platelet-rich plasma.

## Introduction

After anterior cruciate ligament (ACL) reconstruction, two biological mechanisms take place: ligamentization of the intra-articular part of the graft and healing in the bone tunnel.^1^–^5^

Among other processes, in the chronic reparative phase of intratunnel graft healing, chondrification, neo-ossification and proliferative osteoblastic activity are present at about 6 weeks after reconstruction^3^ with the formation of new cortical bone, creating the tunnel wall.

During the physiological cascades of soft tissue healing and bone growth, cellular and hormonal factors play an important role, the most important among them being various growth factors (GF).^5^,^6^ These proteins have a positive effect on fibroblast proliferation and the synthesis of extracellular matrix proteins and therefore on the enhancement of tissue healing.^7^–^9^ Platelet-derived growth factors (PDGFs) particularly enhance the graft incorporation process.^9^

Platelet-rich plasma (PRP), defined as a portion of the plasma fraction of autologous blood having a platelet concentration above the baseline, contains an autologous concentration of platelets and growth factors.^9^,^10^ PRP can be activated with thrombin to create platelet-rich plasma gel. The role of local application of various GF in ACL reconstruction has been analysed by previous studies in animals, where their effect has been evaluated with histological findings or biomechanical tests.^11^–^14^

MRI is because of its excellent contrast resolution^15^,^16^ feasible method for demonstrating the osteosclerosis, where cortical bone is hypointense on all pulse sequences. To the best of our knowledge, no radiological research has yet evaluated the effect of PRP on the formation of cortical bone encircling the tibial tunnel after ACL reconstruction in humans. We found only one study, which assessed the cortication of the tunnel wall, however, only its presence was evaluated with CT, in comparing two different graft fixation screws.^17^ The enhancing effect of PRP and bone GF on bone formation was demonstrated by histological studies in animal models as well as in periodontology.^18^–^25^

Therefore, the aim of presented study was to assess the effect of intraoperatively locally applied PRP, using MRI, for quantitative but noninvasive measurement of the cortical sclerotic bone formation, encircling the tibial tunnel. We hypothesized that PRP promotes the tunnel wall cortical bone (TCB) formation that can be quantitatively assessed by MRI.

## Patients and methods

### Patient selection, surgical technique and platelet gel preparation

The study was designed as a 6-month, single-centre trial, approved by the national ethics committee and was carried out in accordance with the ethical standards laid down in the 1964 Declaration of Helsinki. All patients gave their written informed consent for participation in the study.

The 50 patients included in the study, aged between 18 and 50, were treated for ACL rupture. The main indication for reconstruction was a symptomatic, unstable knee joint due to ACL rupture, assessed by the orthopaedic surgeon. All patients with inflammatory diseases, diabetes mellitus, advanced knee osteoarthrosis (3^rd^ and 4^rd^ degree), previous knee surgery (osteotomies, reconstructive ligament and meniscal procedures and chondral lesions treatment), malignant diseases, allergy to the contrast media, renal diseases and thrombocytopenia were excluded from the study.

All patients undergoing arthroscopic ACL reconstruction were randomized into 2 groups: PRP group comprised 25 patients (15 men, 10 women), and the control group (16 men, 9 women).

All procedures were performed by the same orthopaedic surgeon. In all cases, the standard arthroscopic reconstructive procedure, using the single-incision technique with a double-looped semitendinosus and gracilis tendon graft, was performed. The tunnel sizes in the tibia and femur were matched to the cross-sectional size of the graft and measured 7–9 mm in diameter. The graft was inserted antegrade *via* the tibial and femoral tunnel and fixed with 2 bioabsorbable cross pins (3.2 mm, DePuy Mitek, Massachussets, USA) in the femoral tunnel and with one bioabsorbable interference screw (8–10 mm, DePuy Mitek, Massachusetts, USA) in the tibial tunnel.

The patients in the platelet group received a local application of PRP, which the patients in the control group did not receive. PRP preparation was similar to that previously described.^26^,^27^ During the surgical procedure; autologous blood was obtained and centrifuged. The fraction of PRP was then mixed with activated autologous human thrombin and applied after autograft positioning, into the femoral and tibial tunnels (1 ml in each of them), as well as onto the graft itself (3 ml), where the autologous PRP was formed. An interference screw was inserted after PRP application. All patients were blinded to the treatment with PRP. Both groups followed the same standard rehabilitation protocol.

### Radiological assessment

The MRI was performed in proton density (PD) sequence with fat suppression (TR 2900ms, TE 22 ms, 3 NEX, matrix 320×224, FOV 200, slice thickness 3 mm, 1 mm spacing). The tibial tunnel was examined in the paraxial plane, perpendicular to the tunnel axis. The tibial tunnel is more easily identified than the femoral tunnel on scout images, which facilitates planning and analysis. Examinations were performed one, two and a half and six months after the ACL reconstruction.

At the first examination one month after reconstruction, the slice with the most pronounced TCB between the tibial plateau and the tip of the interference screw, where images were free of volume averaging from the plateau and artefacts from the screw, was chosen for the analysis. In each patient, the same slice was chosen in follow-up assessments.

**FIGURE 1:**
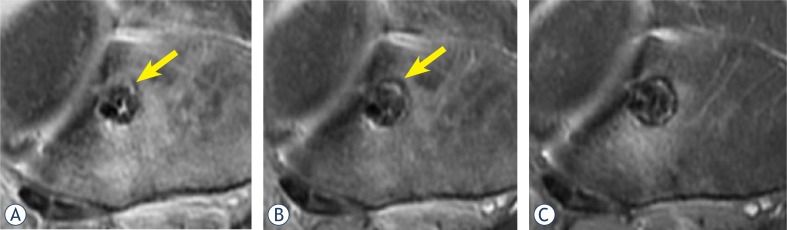
Proton-density weighted fat-suppressed paraxial images just below the tibial plateau from the same patient one (A), two and a half (B) and six (C) months after reconstruction. Because of the perpendicular orientation of slices the cross section of the tibial tunnel was in the rule circular. At the first month (A), only small part of the tunnel wall is sclerotic (estimated to be 10%, arrow). At two and a half months (B) about 20% of the tunnel wall is sclerotic. At six months (C), a thick sclerotic rim encircles estimated 90% of the tunnel. Note also some high signal intensity surrounding the tunnel, representing oedema, which also decreased during the follow-up.

TCB was defined as a clearly hypointense rim of the tibial tunnel wall that was at least 1 mm thick, assessed on paraxial slices. The portion of the tunnel wall circumference, consisting of TCB, was assessed by the consensus of the radiologist (M.R.) and the orthopaedic surgeon (M.V.), rounded off to ten percent (Figure 1). The examiners were blinded to group assignment but not to the time of the procedure.

### Statistical analysis

Numerical data are presented as mean values, while categorical data are expressed as proportions. Differences in TCB between the platelet and control groups were analysed using the Mann-Whitney test. Changes in TCB from the first to the final examination after ACL reconstruction were analysed by the Friedman two-way analysis of variance. As the post-hoc tests, Wilcoxon’s signed ranks tests with Keppels modification of the Bonferroni correction of alpha were used. A P value less than 0.05 were taken to represent statistical significance. Data were analysed using PASW 18 software (SPSS Inc., Chicago, IL, USA).

## Results

20 patients from the control group and 21 patients from the PRP group were available for the follow-up and were analysed, while 9 patients from initial group were lost to follow-up. A comparison of the preoperative parameters of remaining patients in the PRP and the control groups showed that both groups were comparable in gender, age, injury site and body mass index (Table 1).

We observed a gradual increase in the percentage of the tunnel wall consisting of TCB during the follow-up (Table 2). In each group, post-hoc comparisons showed a significant increase in average values of TCB between the three control examinations with increasing postoperative time (p<0.001 in all paired comparisons).

At the first postoperative month we found only small amount of TCB in each group with a nonsignificant difference between the groups (p = 0.928). At two and a half months as well as at six months after surgery, the mean percentage of TCB (Table 2) was significantly higher in the PRP group than in the control group (36.2 *vs* 22.5 and 67.1 *vs* 53.5, p = 0.004 and p = 0.003, respectively).

## Discussion

For ethical reasons, histological evaluation of ACL graft incorporation in humans is impossible, particularly in the bone tunnels. However, MRI is a method of choice for the evaluation of the knee.^28^ Graft healing in the tibial tunnel starts immediately after the operation as an acute inflammatory response with oedema, neutrophils and recruited macrophages present in the tendon bone interface as early as 4 days after surgery.^2^,^3^ At 3 weeks, small vessels appear along with an increased number of osteoblasts on the bone surface. After 6 weeks, the vessels decrease in number and there is a shield-like new bone formation surrounding the graft as well as an increased number of collagen fibers integrating along the tendon.^6^ Sharpey-like fibers, which anchor the fibroproliferative process to the bone, were found at 6 to 12 weeks after reconstruction in a dog model, followed by progressive bone ingrowth.^2^,^3^ The tissue maturation process is finished at about 26 weeks, although this differs among various animals and also between different types of grafts.^1^,^2^,^4^

In the intratunnel graft healing process, incorporation progresses through the formation of a new matrix at the tendon-bone interface. Proliferation of new bone *trabeculae* along the edge of the tunnel is seen as early as three weeks after surgery.^6^ This pattern is not uniform, as some areas exhibit a cartilaginous interface between tendon and bone. The zone of fibrocartilage may persist and represents a form of direct healing by tissue which may undergo enchondral bone formation.^6^ This histological evidence is consistent with our observation of focal areas of sclerosis, representing the TCB, which in the follow-up period in each group expanded and finally fused. Although, on average, two thirds of the tibial wall circumference were sclerotic in the PRPG group at six months, we did not detect complete TCB formation surrounding the entire tunnel in any patient. This could be the consequence of a relatively short follow-up period and is in accordance with the observation of increased osteoblastic activity up to two years after reconstruction.^4^

Several reports of the enhancing effect of PDGF and various other GFs on bone proliferation exist, particularly in periodontology.^11^–^25^ These factors have been shown to enhance murine osteoblast activity and proliferation *in vitro*.^23^*In vivo*, 2-fold increases in uptake of the bone-seeking radiopharmaceutical Technetium 99-MDP, as well as histologically up to a 10-fold increase in new bone and cement were found in dogs.^21^ Locally added PRP accelerated bone healing after mandibular reconstruction in goats considerably.^25^ In humans, significant increase in alveolar bone formation in the process of periodontal regeneration was demonstrated as effects of locally applied PDGF and insulin-like GF.^22^,^24^

Despite the lack of radiological evidence in humans, assessment with MRI in sagittal and coronal slices demonstrated significantly more periosteal and intratunnel bone formation as effects of locally applied combination of bone GF during the ACL reconstruction in rabbits. This was histologically confirmed with more extensive new bone *trabeculae* and cartilage formation at the tendon-bone interface at two and eight weeks after reconstruction and generally more mature tissue at the graft-tunnel interface.^18^ In addition, other histological studies in animal models also showed more new bone formation at this interface as effects of local application of bone morphogenetic protein (BMP)-7 and BMP-2 up to eight weeks after reconstruction.^19^,^20^ Moreover, all of the above-mentioned studies also found higher tensile strength in bone GF groups.

We did not detect differences in the TCB between the groups at one month, which is not in agreement with the above-mentioned animal studies, possibly because of a higher sensitivity with histological analysis, which may detect small changes in the TCB in the first weeks after surgery, when changes are too subtle for observation with MRI. The assessment of joint or even graft stability was not a part of the study protocol which could represent a limitation. However, increased anterior knee stability resulting from PDGF or bone GF has been demonstrated in various studies of humans and animals,^18^–^20^,^27^ although the clinical effect of the PRP is still debated.^26^,^29^ There are several other limitations of the study. We did not evaluate the femoral tunnel because such examination would have to be performed in a different plane, and would therefore significantly prolong the examination. In the tibial tunnel, the analysis of additional neighbouring slices could be more accurate, but also time-consuming, therefore in each patient the same slice was meticulously selected for analysis in all follow-up examinations. Owing to superior spatial resolution with even thinner slices, CT examination could be possibly more accurate, but at the cost of significant radiation exposure, important particularly in this young study population. We did not perform preoperative MRI, nor did we evaluate for lower degrees of osteoarthrosis and concurrent procedures during arthroscopy, as well as injury chronicity and possible drugs intake like NSAR and corticosteroids. All these factors could influence the inflammatory response in the healing process, which is, however, rather questionable in the intratunnel region compared with intra-articular inflammation.

In conclusion, the results of our study demonstrate that formation of focal areas of sclerotic cortical bone with subsequent fusion into a thick tibial tunnel wall is a part of the ACL graft incorporation process that can be quantitatively assessed by MRI. Furthermore, we observed that local application of PRP results in enhanced cortical bone formation encircling the tibial tunnel at 2.5 and 6 months but not 1 month after ACL reconstruction.

## Figures and Tables

**TABLE 1:** Preoperative data on injured patients

**Characteristic**	**Control group (n=20)**	**PRP group (n=21)**	**p-value for differences between groups**
Men, n (%)	15 (75)	13 (62)	0.505
Age, years	32.6±12.3	37.2±8.4	0.112
Injured knee, n (%)			1.000
Right	12 (60%)	12 (57%)	
Left	8 (40%)	9 (43%)	
BMI	24.5±2.1	26.5±4.1	0.078

Numerical data are expressed as mean ± standard deviation; BMI = body mass index

**TABLE 2:** Percentage of tibial tunnel wall cortical bone in both groups at follow-up examinations. Values are given as means (95% confidence interval)

**Period from surgery (months)**	**Control group (n=20)**	**PRP group (n=21)**	**p-values for differences between groups**
1	5.0 (1.8–8.2)	6.7 (1.8–11.5)	0.928
2.5	22.5 (17.3–27.7)	36.2 (28.7–43.7)	0.004
6	53.5 (47.0–60.0)	67.1 (61.0–73.3)	0.003
